# Engineering of l-threonine and l-proline biosensors by directed evolution of transcriptional regulator SerR and application for high-throughput screening

**DOI:** 10.1186/s40643-024-00837-6

**Published:** 2025-01-19

**Authors:** Wei Pu, Jinhui Feng, Jiuzhou Chen, Jiao Liu, Xuan Guo, Lixian Wang, Xiaojia Zhao, Ningyun Cai, Wenjuan Zhou, Yu Wang, Ping Zheng, Jibin Sun

**Affiliations:** 1 Key Laboratory of Engineering Biology for Low-carbon Manufacturing, Tianjin Institute of Industrial Biotechnology, Chinese Academy of Sciences, Tianjin, 300308 China; 2 Key Laboratory of Regional Characteristic Agricultural Resources, College of Life Sciences, Neijiang Normal University, Neijiang, 641100 China; 3 Haihe Laboratory of Synthetic Biology, Tianjin, 300308 China; 4 National Center of Technology Innovation for Synthetic Biology, Tianjin, 300308 China; 5 University of Chinese Academy of Sciences, Beijing, 100049 China; 6 College of Biotechnology, Tianjin University of Science and Technology, Tianjin, 300457 China

**Keywords:** l-Threonine and l-proline biosensor, Transcriptional regulator SerR, Directed evolution, High-throughput screening, l-Homoserine dehydrogenase, γ-Glutamyl kinase

## Abstract

Amino acids are important bio-based products with a multi-billion-dollar market. The development of efficient high-throughput screening technologies utilizing biosensors is essential for the rapid identification of high-performance amino acid producers. However, there remains a pressing need for biosensors that specifically target certain critical amino acids, such as l-threonine and l-proline. In this study, a novel transcriptional regulator-based biosensor for l-threonine and l-proline was successfully developed, inspired by our new finding that SerE can export l-proline in addition to the previously known l-threonine and l-serine. Through directed evolution of SerR (the corresponding transcriptional regulator of SerE), the mutant SerR^F104I^ which can recognize both l-threonine and l-proline as effectors and effectively distinguish strains with varying production levels was identified. Subsequently, the SerR^F104I^-based biosensor was employed for high-throughput screening of the superior enzyme mutants of l-homoserine dehydrogenase and γ-glutamyl kinase, which are critical enzymes in the biosynthesis of l-threonine and l-proline, respectively. A total of 25 and 13 novel mutants that increased the titers of l-threonine and l-proline by over 10% were successfully identified. Notably, six of the newly identified mutants exhibited similarities to the most effective mutants reported to date, indicating the promising application potential of the SerR^F104I^-based biosensor. This study illustrates an effective strategy for the development of transcriptional regulator-based biosensors for amino acids and other chemical compounds.

**Supplementary Information:**

The online version contains supplementary material available at 10.1186/s40643-024-00837-6.

## Introduction

Amino acids are the basic building blocks of various biologically functional macromolecules, particularly proteins, and have been widely applied in animal feed, food, pharmaceutical, cosmetic, and daily chemical industries. Amino acid industry is one of the pillar industries of biomanufacturing, the worldwide market for amino acids reached an overall 10.3 million tons, with gross sales of $28 billion in 2021. The global amino acid market is expected to expand at a compound annual growth rate of 6.76% in the next decade (Tuo et al. 2023). Microbial fermentation is considered as an economical, efficient, and environmentally friendly method for amino acid production, contributing to approximately 80% of the global amino acids yield (Sanchez et al. 2018; Wendisch 2014). To improve the industrial production level of amino acids, microbial cell factories are developed by random mutation, metabolic engineering, and high-throughput screening (Han et al. 2020; Zhang et al. 2021). Besides, the key functional elements such as rate-limiting enzymes, transporters, and transcriptional regulators involved in amino acid biosynthesis and transport are also extensively engineered by rational design and directed evolution (Wang et al. 2019b). Therefore, the development of effective high-throughput screening methods for amino acid producing strains and key enzymes is important.

Genetically encoded biosensors are the vital components of synthetic biology and metabolic engineering, as they are regarded as powerful devices for dynamic regulation of metabolic pathways and high-throughput screening of desirable phenotypes for improving the performance of industrial microorganisms (Deng et al. 2022; Yu et al. 2023; Zeng et al. 2020). Until now, different types of amino acid biosensors have been developed, such as biosensors based on the transcriptional regulator (Binder et al. 2012; Xu et al. 2020), riboswitch (Sudarsan et al. 2003; Zhou and Zeng 2015a, b) Förster resonance energy transfer (FRET) (Frommer et al. 2009), and translation machinery (Guo et al. 2023; Sun et al. 2020; Zheng et al. 2018). Biosensors for alkaline amino acids (l-lysine, l-arginine, and l-histidine) (Binder et al. 2012; Jiang et al. 2023), branch-chain amino acid (l-valine, l-leucine, and l-isoleucine) and l-methionine (Mustafi et al. 2012; Sun et al. 2020), aromatic amino acids (l-tryptophan, l-tyrosine, and l-phenylalanine) (Liu et al. 2017b, 2021), l-glycine (Zhou et al. 2019), l-serine (Binder et al. 2012), and l-cysteine (Gao et al. 2022) have been developed and widely applied in the evolution and screening of amino acid producing strains and related enzymes (Binder et al. 2012; Della Corte et al. 2020; Han et al. 2020; Kortmann et al. 2019; Liu et al. 2017b, 2021, 2022b; Mahr et al. 2015; Pu et al. 2023; Schendzielorz et al. 2014; Stella et al. 2021; Zhang et al. 2018), dynamic regulation of amino acid biosynthetic pathways (Tan et al. 2020), and live cell imaging (Mustafi et al. 2014; Vasdekis and Stephanopoulos 2015). However, biosensors for some amino acids with important applications and large market demands, such as l-threonine, l-proline, l-glutamate, and l-aspartate, remain unavailable. For example, l-threonine is an essential amino acid that cannot be synthesized by humans and animals and has the third largest market size as a feed additive (Fang et al. 2020; Wendisch 2020). l-Proline, the only proteinogenic amino acid with a secondary amine, is a high-value amino acid with applications in medicine and health care industry and has large potential for use as a feed additive (Wendisch 2014). Therefore, it is of great significance to develop l-threonine and l-proline biosensors for high-throughput screening of l-threonine and l-proline hyper-producing strains and key enzymes.

**Fig. 1:**
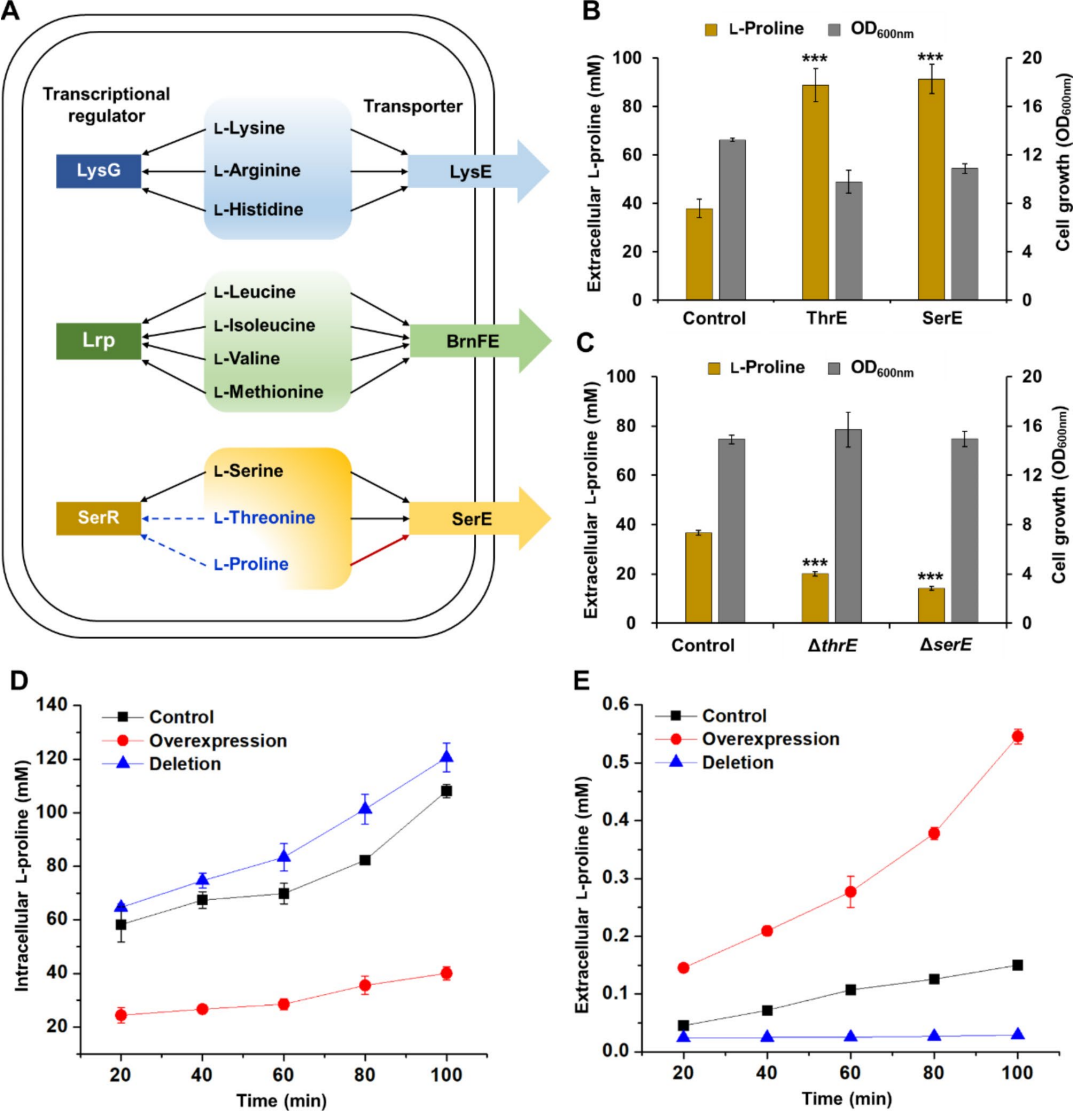
Characterization of the transporting function of the exporter SerE for l-proline. **(A)** The correlation between the substrate spectrum of amino acid transporters and the corresponding transcriptional regulators. The black solid arrow represents the amino acid can be recognized by the transporter or transcriptional regulator. The blue dotted arrow represents the amino acid is predicted to be recognized by the transcriptional regulator. The red arrow means the amino acid is demonstrated to be recognized by the exporter in this study. **(B)** Effects of overexpression of exporters ThrE and SerE on l-proline production. The *thrE* and *serE* genes of *C. glutamicum* were individually overexpressed in the plasmid pEC-XK99E under the control of an isopropyl-β-d-thiogalactopyranoside (IPTG)-inducible promoter P_*trc*_, and transformed into an l-proline-producing strain *C. glutamicum* ATCC 13032 (ProB^G149K^) (designated as Pro1). Control, ThrE, and SerE represent strains Pro1 (pEC-XK99E), Pro1 (pEC-XK99E-*thrE*), and Pro1 (pEC-XK99E-*serE*), respectively. Strains were cultivated in TSB medium with 10 g/L urea for 18 h, and samples were used for l-proline assay (Liu et al. 2022a). Values and error bars reflect the mean ± s.d. of three biological replicates (*n* = 3). ***P < 0.001, student’s two-tailed *t*-test. **(C)** Effects of inactivation of exporters ThrE and SerE on l-proline production. Control, Δ*thrE*, and Δ*serE* represent strains *C. glutamicum* Pro1, Pro1 (Δ*thrE*), and Pro1 (Δ*serE*), respectively. Values and error bars reflect the mean ± s.d. of three biological replicates (*n* = 3). ***P < 0.001, student’s two-tailed *t*-test. (D/E) Changes in intracellular **(D)** and extracellular **(E)** l-proline concentrations upon Pro-Gly dipeptide addition. Control, *C. glutamicum* ATCC 13032. Deletion, *C. glutamicum* Δ*serE*. Overexpression, *C. glutamicum* (pEC-XK99E-*serE*). The experimental procedures of dipeptide uptake and amino acid export assay were conducted according to the previously reported method (Liu et al. 2022a). Extracellular and intracellular l-proline concentrations were quantified according to the method based on the acid-ninhydrin reaction (Bates et al. 1973).Values and error bars reflect the mean ± s.d. of three biological replicates (*n* = 3)

Most of reported amino acid biosensors were constructed based on the regulatory machinery of amino acid transport, such as the transcriptional regulator LysG regulating alkaline amino acid exporter LysE (Binder et al. 2012) and the Lrp regulating branch-chain amino acid exporter BrnFE (Lange et al. 2012). The findings suggest a hypothesis that if certain of compounds (e.g. l-lysine, l-arginine, and l-histidine) are accepted by a transporter (e.g. LysE), they are very probably also accepted by the corresponding transcriptional regulator (e.g. LysG) (Fig. 1A). ThrE (Cgl2622) of *Corynebacterium glutamicum* has been identified as the exporter of l-serine and l-threonine (Simic et al. 2001), and l-proline (Liu et al. 2022a). However, the transcriptional regulator for ThrE has not been identified yet. SerE (Cgl0605) of *C. glutamicum* is capable of excreting l-serine (Binder et al. 2012) and l-threonine (Zhang et al. 2020b), which is transcriptionally regulated by SerR (Cgl0606), an LysR-type transcriptional regulator (LTTR). SerR could sense the intracellular l-serine and activate the expression of SerE for l-serine excretion (Binder et al. 2012). SerE (l-serine and l-threonine) shares overlapped substrate spectrum with ThrE (l-serine, l-threonine, and l-proline). Therefore, inspired by the regulatory machinery of amino acid transport, it is speculated that SerR may have the potential to recognize l-threonine and l-proline besides l-serine as its effectors.

In this study, SerE was firstly demonstrated as the exporter of l-proline besides l-threonine and l-serine. Although the corresponding wild type SerR was found specifically responding to l-serine instead of l-proline or l-threonine, directed evolution of SerR generated a SerR^F104I^ mutant capable of responding to l-threonine and l-proline. By using the SerR^F104I^ mutant as a sensory protein and the enhanced yellow fluorescent protein (eYFP) as an easily detectable reporter, an l-threonine and l-proline whole-cell biosensor pSerR^F104I^ was successfully developed for the first time. Subsequently, the biosensor was applied to high-throughput screen key enzymes involved in l-threonine and l-proline biosynthesis (l-homoserine dehydrogenase, Hom and γ-glutamyl kinase, ProB). This study provides a new strategy for selecting a suitable transcriptional regulator to change its effector specificity for developing new biosensors for amino acids and other chemicals.

## Results and discussion

### Characterization of the transporting function of the exporter SerE for l-proline

To test our hypothesis that SerE may also serve as an l-proline exporter besides l-serine and l-threonine, the exporter SerE, as well as a positive control of the l-proline exporter ThrE, was individually overexpressed in an l-proline-producing strain *C. glutamicum* ATCC 13032 (ProB^G149K^) (designated as Pro1) and l-proline production was examined because enhancing excretion of the target molecule has been proven beneficial to bioproduction (Liu et al. 2022a). Overexpression of ThrE increased the l-proline titer by 2.34-fold, while overexpression of SerE displayed similar improvement in l-proline production (2.41-fold) (Fig. 1B). Moreover, previous studies have demonstrated that inactivation of exporters reduced the extracellular accumulation of amino acids (Liu et al. 2022a; Zhang et al. 2020b). Therefore, the exporters ThrE and SerE were deleted in the l-proline-producing strain Pro1, respectively. The results showed that the deletion of ThrE and SerE significantly decreased the extracellular l-proline level, which is 1.82-fold and 2.57-fold lower than that of the control strain Pro1, respectively (Fig. 1C). These results manifest that SerE can also mediate the export of l-proline like ThrE.

To further verify the function of SerE as an l-proline exporter, dipeptide uptake and amino acid export assay was conducted. *serE* was deleted and overexpressed in *C. glutamicum* ATCC 13032, respectively. Upon the addition of Pro-Gly dipeptide, the *serE*-deleted mutant showed higher intracellular l-proline level and dramatically decreased l-proline export rate (Fig. 1D and E). Conversely, overexpression of *serE* in a plasmid with IPTG-inducible promoter P_*trc*_ largely decreased intracellular l-proline concentration but accelerated l-proline export (Fig. 1D and E). These results suggest that SerE is indeed as an l-proline exporter.

### Directed evolution of SerR for recognizing l-threonine and l-proline

**Fig. 2:**
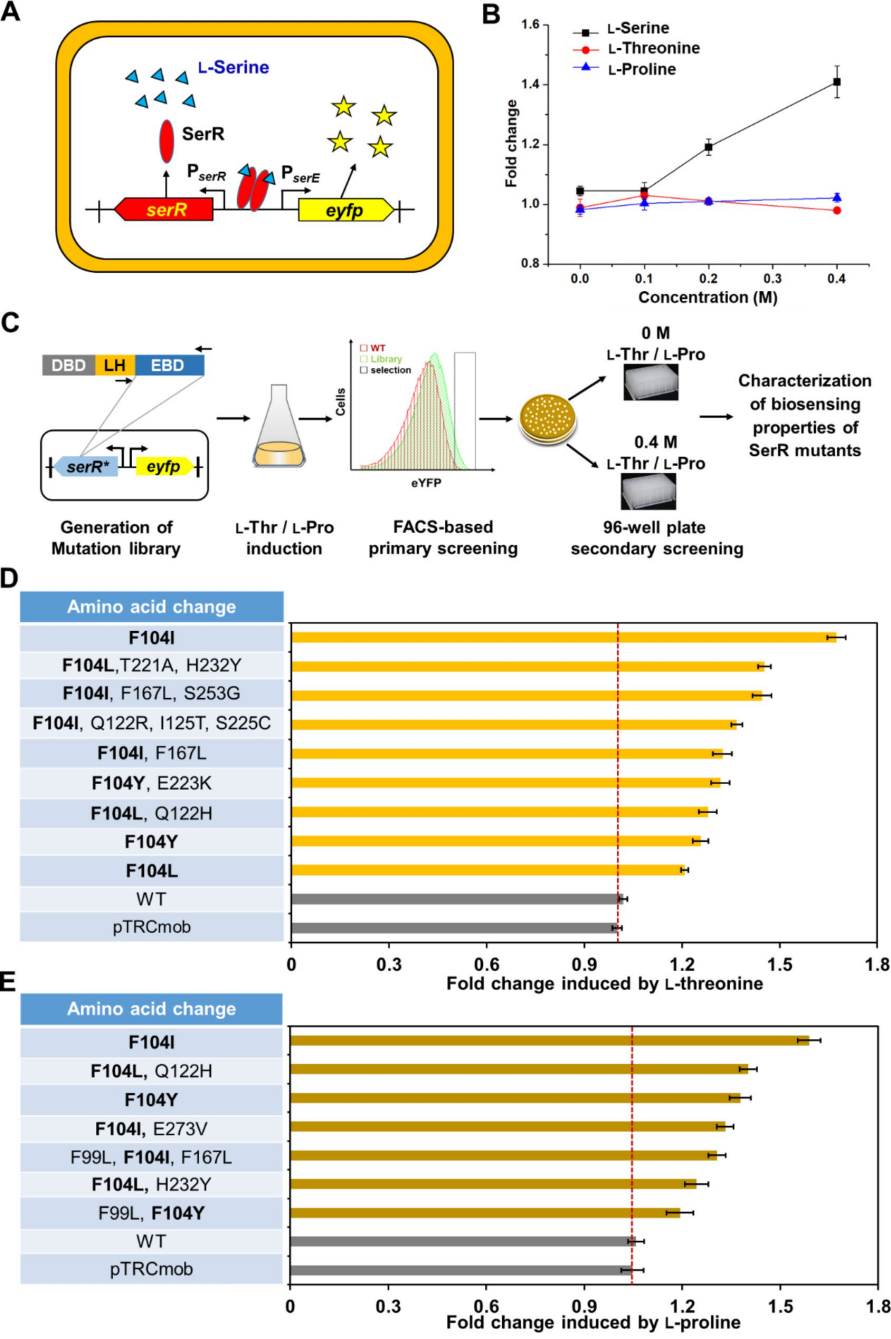
Directed evolution of SerR for sensing l-threonine and l-proline. **(A)** The schematic illustration of the SerR-based whole-cell biosensor pSerR^WT^ for transforming l-serine concentration into eYFP signal in *C. glutamicum*. **(B)** Dose-response curves of the pSerR^WT^ biosensor responding to different amino acids. Fold change represents the fluorescence output of *C. glutamicum* harboring the pSerR^WT^ divided by that of *C. glutamicum* harboring the pTRCmob empty plasmid. Strains were cultivated in modified CGXII medium (Pu et al. 2023) supplemented with different concentrations of l-serine, l-threonine, or l-proline for 12 h. Values and error bars reflect the mean ± s.d. of three biological replicates (*n* = 3). **(C)** Workflow of directed evolution of SerR and fluorescence activated cell sorting (FACS)-based screening of desired mutants. DBD, DNA-binding domain. LH, linker helix. EBD, effector-binding domain. Strains were cultivated in modified CGXII medium with or without adding 0.4 M l-threonine (or l-proline) for 12 h, and subjected to FACS analysis. The sorting gate is indicated with a black box in the histogram. Mutants with higher eYFP outputs in the presence of 0.4 M l-threonine (or l-proline) but lower basal eYFP outputs without l-threonine induction were selected for further characterization. **(D)** Screened SerR mutants responding to l-threonine. **(E)** Screened SerR mutants responding to l-proline. pTRCmob and WT represent *C. glutamicum* ATCC 13032 harboring the empty plasmid pTRCmob and pSerR^WT^, respectively. Strains were cultivated in modified CGXII medium supplemented with or without 0.4 M l-threonine (or l-proline) for 12 h, and used for detection of the eYFP fluorescence. Fold change represents the fluorescence output of strain *C. glutamicum* ATCC 13032 harboring the pSerR^WT^ (or its mutants) with adding inducer divided by that of the same strain without adding inducer. Values and error bars reflect the mean ± s.d. of three biological replicates (*n* = 3)

To test whether the transcriptional regulator SerR could recognize l-threonine and l-proline besides l-serine as its effectors, a whole-cell biosensor dubbed pSerR^WT^ was developed using SerR as a sensory protein and eYFP as an easily detectable reporter under the control of the cognate *serE* promoter (Fig. 2A). Then, the responsive activities of pSerR^WT^ to l-serine, l-threonine, and l-proline were determined. With the increase of exogenous addition of l-serine, elevated eYFP signals were observed, whereas no eYFP signals were produced upon addition of l-threonine or l-proline (Fig. 2B). These results suggest that the pSerR^WT^ biosensor can only respond to l-serine and transform its intracellular level into visual optical signal.

Since the wild-type SerR cannot recognize l-threonine and l-proline, we speculated that this may be due to the possibility that SerR had narrowed its effector spectrum over the course of evolution. Therefore, we performed directed evolution of SerR to reversely engineer its effector spectrum. Since directed evolution targeting the effector binding domain (EBD) of transcriptional regulators is an effective strategy to switch the effector specificity and recognize new effectors (Li et al. 2017, 2022; Snoek et al. 2020; Taylor et al. 2016; Wu et al. 2022), the EBD of SerR was located by structure modeling of SerR using the AlphaFold2 (Jumper et al. 2021; Varadi et al. 2022). The amino acid residues of L87 to E304 are suggested as the EBD of SerR (Fig. S1). To engineer the effector spectrum of SerR and maintain its DNA binding activity, a random mutation library targeting only the EBD was constructed by error-prone PCR. The plasmid library harboring SerR mutants was then transformed into *C. glutamicum* and screened for desired mutants with enhanced eYFP outpouts responding to l-threonine or l-proline by fluorescence activated cell sorting (FACS) (Fig. 2C).

After the first round of the FACS-based screening, 92 colonies were randomly selected and cultivated in 96-well plates for a second round of screening. For screening with addition of l-threonine, 28 out of the 92 colonies showed significantly increased eYFP outputs than the wild-type biosensor pSerR^WT^ in the presence of 0.4 M l-threonine as an inducer. Plasmid sequencing of the 28 colonies revealed 9 kinds of SerR mutants with different amino acid sequences, all of which had an amino acid substitution at the F104 residue (Fig. 2D). The eYFP output of the best SerR^F104I^ mutant upon l-threonine induction was 1.68-fold higher than that in the absence of l-threonine. When the library was screened with the induction by l-proline, 7 different SerR mutants capable of responding to l-proline were obtained (Fig. 2E), and the mutant SerR^F104I^ showed the highest fluorescence output upon l-proline induction (1.59-fold higher than the control without l-proline). Interestingly, plasmid sequencing shows that all the mutants also have an amino acid substitution at the F104 residue (Fig. 2E). The results suggests that F104 may function as a key residue controlling the effector recognition of SerR.

### Characterization of the mutant SerR^F104I^

**Fig. 3:**
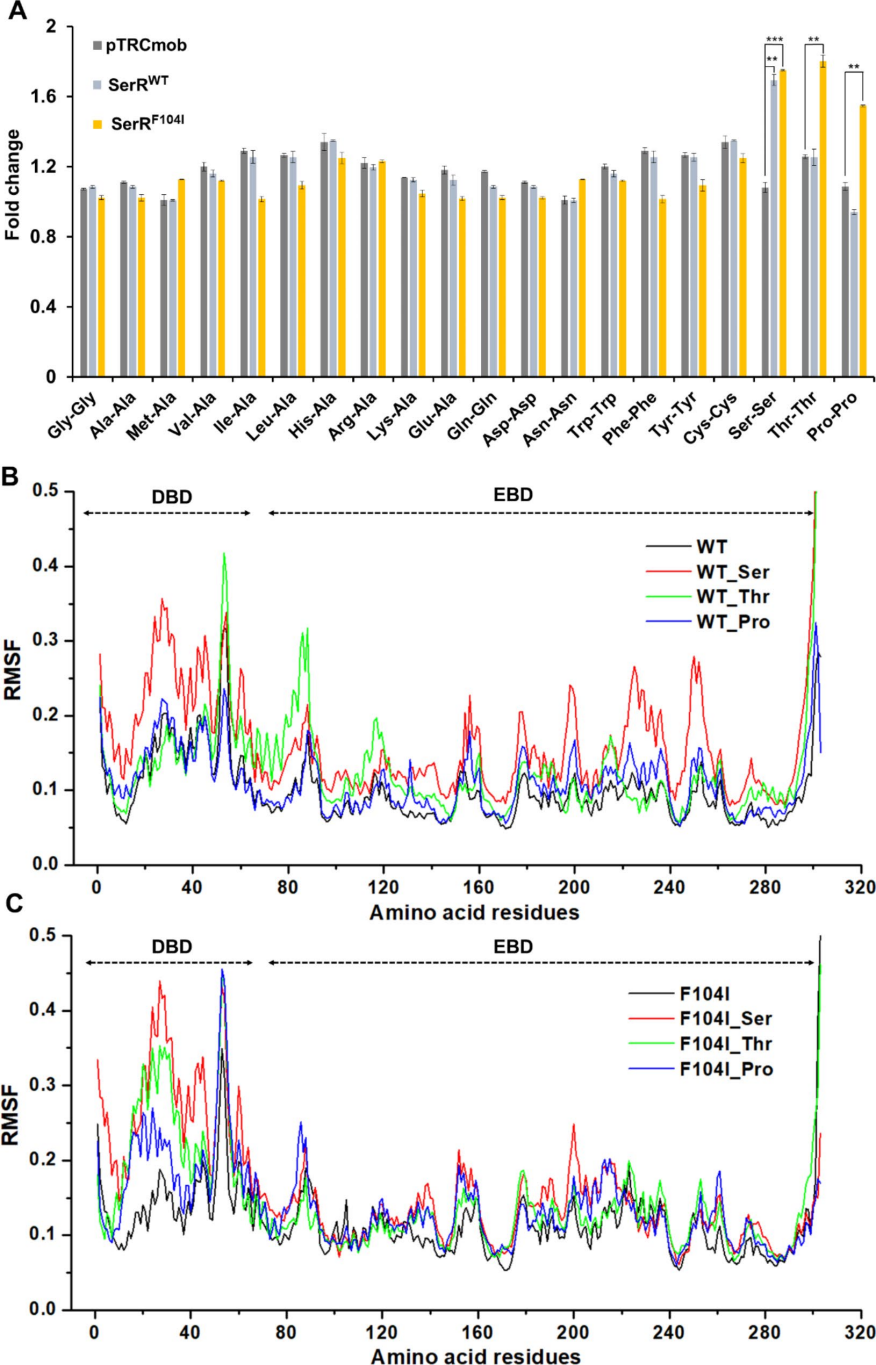
Characterization of the effector specificity of SerR^F104I^. **(A)** Response of the wild-type SerR and mutant SerR^F104I^ to dipeptides of different amino acids. pTRCmob, SerR^WT^, and SerR^F104I^ represent *C. glutamicum* ATCC 13032 harboring the empty plasmid pTRCmob, pSerR^WT^, and pSerR^F104I^, respectively. Strains were cultivated in CGXII medium supplemented with 3 mM amino acid dipeptides for 12 h, and used for detection of eYFP fluorescence and cell growth (OD_600nm_). Fold change represents the fluorescence output of strain *C. glutamicum* ATCC 13032 harboring the pSerR^WT^ (or pSerR^F104I^) with adding 3 mM dipeptide divided by that of the same strain without adding inducer. Strains were cultivated in CGXII medium with 50 g/L glucose and 3 mM dipeptide for 12 h. Values and error bars reflect the mean ± s.d. of three biological replicates (*n* = 3). ***P < 0.001, **P < 0.01, student’s two-tailed *t*-test. **(B)** MD simulation of the wild-type SerR in the absence or presence of effectors. **(C)** MD simulation of the SerR^F104I^ mutant in the absence or presence of effectors. The SerR mutant structures and complexes with l-serine, l-threonine, and l-proline were generated by the Discovery Studio 4.1 using mutagenesis tool and flexible docking tool, respectively. MD simulations were performed by GROMACS using the Amber14SB force field (Abraham et al. 2015; Páll et al. 2015). All the complexes were solvated in a cubic TIP3P water box with periodic boundary conditions. Visual molecular dynamics (VMD) was employed for visualization and analyses the trajectory (Humphrey et al. 1996)

The mutant SerR^F104I^ could respond to l-threonine and l-proline, suggesting that the F104I mutation changed the effector specificity of SerR. To evaluate the ability of SerR^F104I^ to recognize various amino acids, a range of dipeptides was employed, which were sufficiently hydrolyzed into dissociative amino acids within the cells. Results showed that the mutant SerR^F104I^ only responded to l-threonine, l-proline, and l-serine and could not recognize other amino acids. Meanwhile, the wild-type SerR only responded to l-serine (Fig. 3A). Since SerR is classified as a LTTR, we conducted a comparative analysis of the published structures of LTTRs and observed that the effectors are bound within a similar pocket. Subsequently, molecular docking studies were performed for the transcriptional regulator SerR in conjunction with the effector l-serine. The analysis revealed that the distance between residue F104 and l-serine exceeds 8 Å (Fig. S2). This indicates that the F104 site is located at a considerable distance from the effector binding site of SerR. Consequently, it is hypothesized that mutations in the F104 residue may influence the effector specificity of SerR through an indirect mechanism.

Molecular dynamics (MD) simulation was then applied to further resolve the differences in structural dynamics characteristics between the wild-type SerR and SerR^F104I^ mutant. Upon the binding of an effective effector, the transcriptional regulator should have a conformational change, especially the DBD that binds DNA, which will lead to increased root mean square fluctuation (RMSF). MD simulation suggests that RMSF of the DBD of wild-type SerR in the presence of l-serine is significantly higher than that without l-serine, suggesting the conformational change triggered by l-serine binding. In the presence of l-threonine or l-proline, no such changes in RMSF were observed, which was consistent with the fact that wild type SerR did not recognize l-threonine or l-proline (Fig. 3B). However, l-serine, l-threonine, and l-proline could all cause significant changes in the RMSF of SerR^F104I^ mutant (Fig. 3C). These results suggest that the SerR^F104I^ mutant binding with l-threonine and l-proline exhibited higher flexibility in the DBD than the wild-type SerR. The dynamical characteristics of SerR^F104I^ were consistent with its regulatory properties.

### High-throughput screening of Hom variants by using the SerR^F104I^-based whole-cell biosensor

An l-threonine and l-proline whole-cell biosensor dubbed pSerR^F104I^ was developed using the mutant SerR^F104I^ as a sensory protein and eYFP as an easily detectable reporter. Furthermore, we tested the performance of the whole-cell biosensor pSerR^F104I^ in high-throughput screening of key enzymes in l-threonine and l-proline biosynthesis.

**Fig. 4:**
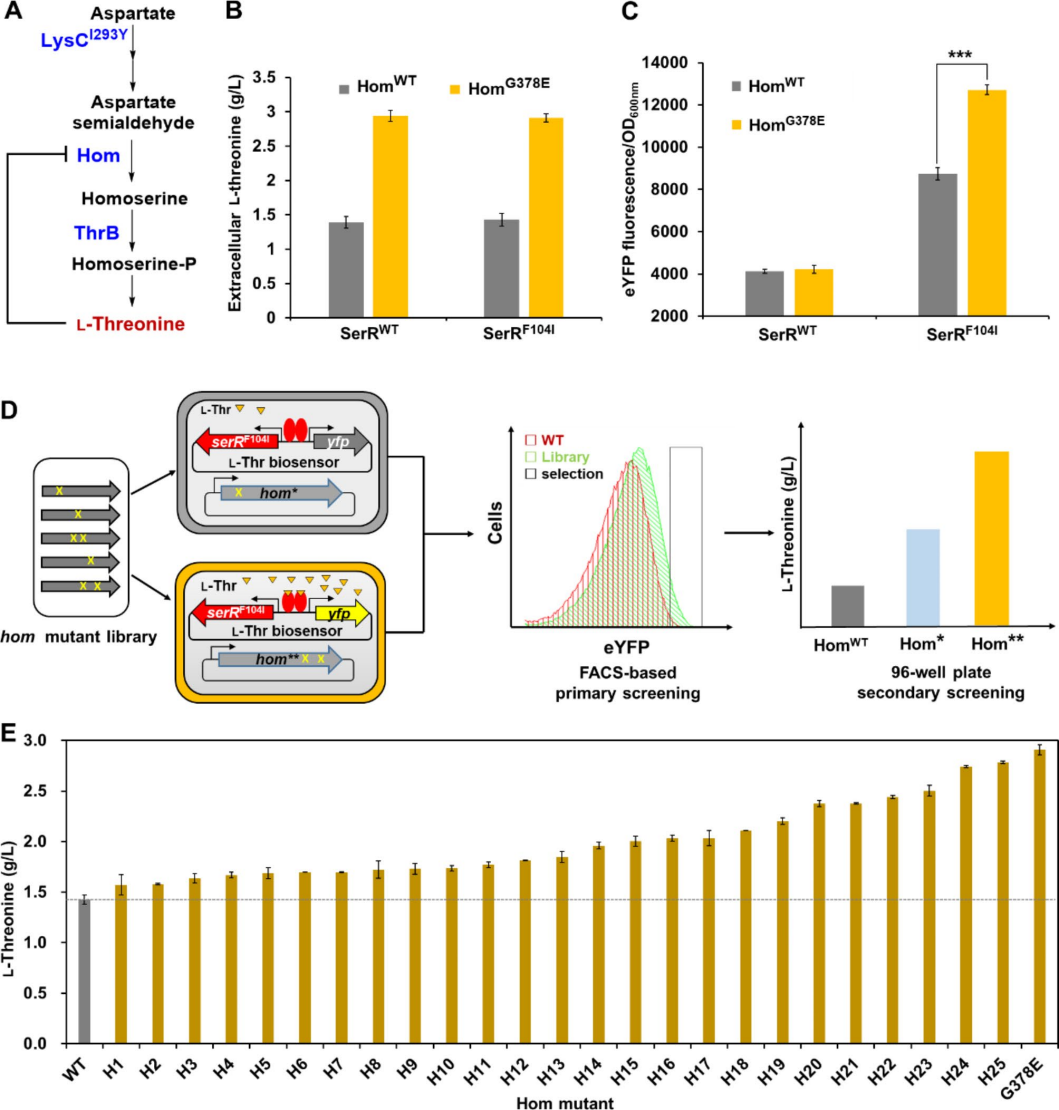
FACS-based screening of Hom mutants facilitating l-threonine production. **(A)** Biosynthetic pathway of l-threonine in *C. glutamicum*. The key enzyme l-aspartate kinae (LysC), l-homoserine dehydrogenase (Hom), and l-homoserine kinase (ThrB) are highlighted in blue. The feedback inhibition of Hom by l-threonine is indicated with black line. **(B)** Extracellular accumulation of l-threonine. Hom^WT^ and Hom^G378E^ represent strain *C. glutamicum* Thr1 (pSerR^WT^-P_11F_-*thrB*) or Thr1 (pSerR^F104I^-P_11F_-*thrB*) transformed with the plasmid pXMJ19 overexpressing the wild-type Hom and mutant Hom^G378E^, respectively. Extracellular l-threonine titer was measured after 24 h cultivation in modified CGXII medium with 0.1 mM IPTG. The concentration of l-threonine was quantified using a high-performance liquid chromatograph (HPLC) method (Wang et al. 2018). **(C)** eYFP fluorescence signals of SerR^WT^- and SerR^F104I^-based biosensors with *C. glutamicum* strains overexpressing Hom^WT^ and Hom^G378E^. Strains were cultivation in modified CGXII medium with 0.1 mM IPTG for 12 h, and used for the detection of eYFP fluorescence. Values and error bars reflect the mean ± s.d. of three biological replicates (*n* = 3). ***P < 0.001, student’s two-tailed *t*-test. **(D)** Workflow of high-throughput screening of Hom mutants based on the l-threonine biosensor. **(E)** Characterization of the isolated Hom mutants. The wild-type Hom and isolated mutants were overexpressed in strain *C. glutamicum* Thr1 (SerR^F104I^-P_11F_-*thrB*) to evaluate l-threonine production. The reported Hom^G378E^ mutant was used as a positive control. Strains were cultivated in modified CGXII medium with 0.1 mM IPTG for 24 h. Values and error bars reflect the mean ± s.d. of three biological replicates (*n* = 3)

To test whether the biosensor pSerR^F104I^ allows to distinguish cells with different l-threonine production capabilities, several l-threonine-producing strains were constructed. Firstly, I293Y mutation was introduced into l-aspartate kinase (LysC) to release the feedback inhibition by l-lysine and l-threonine, resulting in strain *C. glutamicum* (LysC^I293Y^) (Fig. 4A) (Zheng et al. 2020). To avoid accumulation of intermediate l-homoserine, *thrB* encoding l-homoserine kinase was constitutively overexpressed in the biosensor plasmid. Previous studies have demonstrated that Hom is also seriously inhibited by l-threonine (Fig. 4A) (Reinscheid et al. 1991), and overexpression of Hom mutants desensitized to feedback inhibition can enhance l-threonine production (Colón et al. 1995). Then, the chromosomal *hom* gene was knocked out and Hom or Hom^G378E^ mutants was overexpressed through an independent plasmid, resulting in strains Thr1 (pSerR^WT^-P_11F_-*thrB*, pXMJ19-*hom*), Thr1 (pSerR^WT^-P_11F_-*thrB*, pXMJ19-*hom*^G378E^), (pSerR^F104I^-P_11F_-*thrB*, pXMJ19-*hom*), and Thr1 (pSerR^F104I^-P_11F_-*thrB*, pXMJ19-*hom*^G378E^). Fermentation experiments conducted in 96-well plates demonstrated that overexpression of the wild-type Hom produced only 1.43 g/L l-threonine, while overexpression of the reported mutant Hom^G378E^ increased the titer of l-threonine by 2.03-fold (2.91 g/L) (Fig. 4B) (Reinscheid et al. 1991). The SerR^WT^-based biosensor pSerR^WT^ produced similar and low eYFP outputs for strains expressing the wild-type Hom and Hom^G378E^ mutant, while the SerR^F104I^-based biosensor pSerR^F104I^ showed 1.46-fold higher eYFP outputs in the strain overexpressing Hom^G378E^ compared with the strain overexpressing the wild-type Hom (Fig. 4C). These results suggest that the SerR^F104I^-based biosensor can respond to l-threonine and distinguish strains with different l-threonine production levels.

Finally, we demonstrated the applications of the SerR^F104I^-based biosensor in high-throughput screening of Hom mutants improving l-threonine production (Fig. 4D). Firstly, a random mutation library of *hom* was constructed by error-prone PCR and cloned into pXMJ19 plasmid. The plasmid library was transformed into *C. glutamicum* Thr1 (pSerR^F104I^-P_11F_-*thrB*). Then, the resultant 1.9 × 10^5^ transformants were collected and cultivated for 6 h, before they were subjected to FACS analysis. The strain harboring a pXMJ19 plasmid overexpressing the wild-type Hom was used as a control. Approximately 0.07% of cells with the highest fluorescence in the library were sorted out, while the portion for the control group was 0.02%, suggesting Hom mutants enabling l-threonine production existed in the library (Fig. S3). Total 92 colonies sorted by FACS were cultivated in 96-well plates for a second round of screening. Among them, 58 strains showed obviously higher eYFP fluorescence than the control strain. Plasmid sequencing of the 58 strains revealed 25 kinds of Hom mutants with different amino acid substitutions. Overexpression of these mutants obtained higher l-threonine titers than the strain expressing wild-type Hom (Fig. 4E and Table S3). The best two mutants Hom^M371I^ and Hom^E134V, G174S, N318S, H342D^ showed over 90% improvement of l-threonine titers compared with the wild-type Hom, which are similar with the positive control Hom^G378E^ (Reinscheid et al. 1991). The results demonstrate that the l-threonine whole-cell biosensor pSerR^F104I^ is an effective tool for high-throughput screening of key enzymes for l-threonine production.

Our recently study obtained some Hom mutants (A381V, A384D, and I397V) enabled the overproduction of l-threonine from the site-saturation mutagenesis libraries of Hom via a growth-coupled screening method based on resistance to l-threonine analog (Liu et al. 2023). All these mutation sites located at the tetramer interface and were predicted to play essential roles in maintaining the tetramer structure and feedback inhibition of l-threonine. In this study, some positive mutants with new mutation sites (such as M371I, D445G, S385N, and A358T) were screened. Interestingly, some of these sites were not included in the tetramer interface. The enzymatic properties of these new mutants will be further investigated in future studies.

### High-throughput screening of ProB variants by using the SerR^F104I^-based whole-cell biosensor

**Fig. 5:**
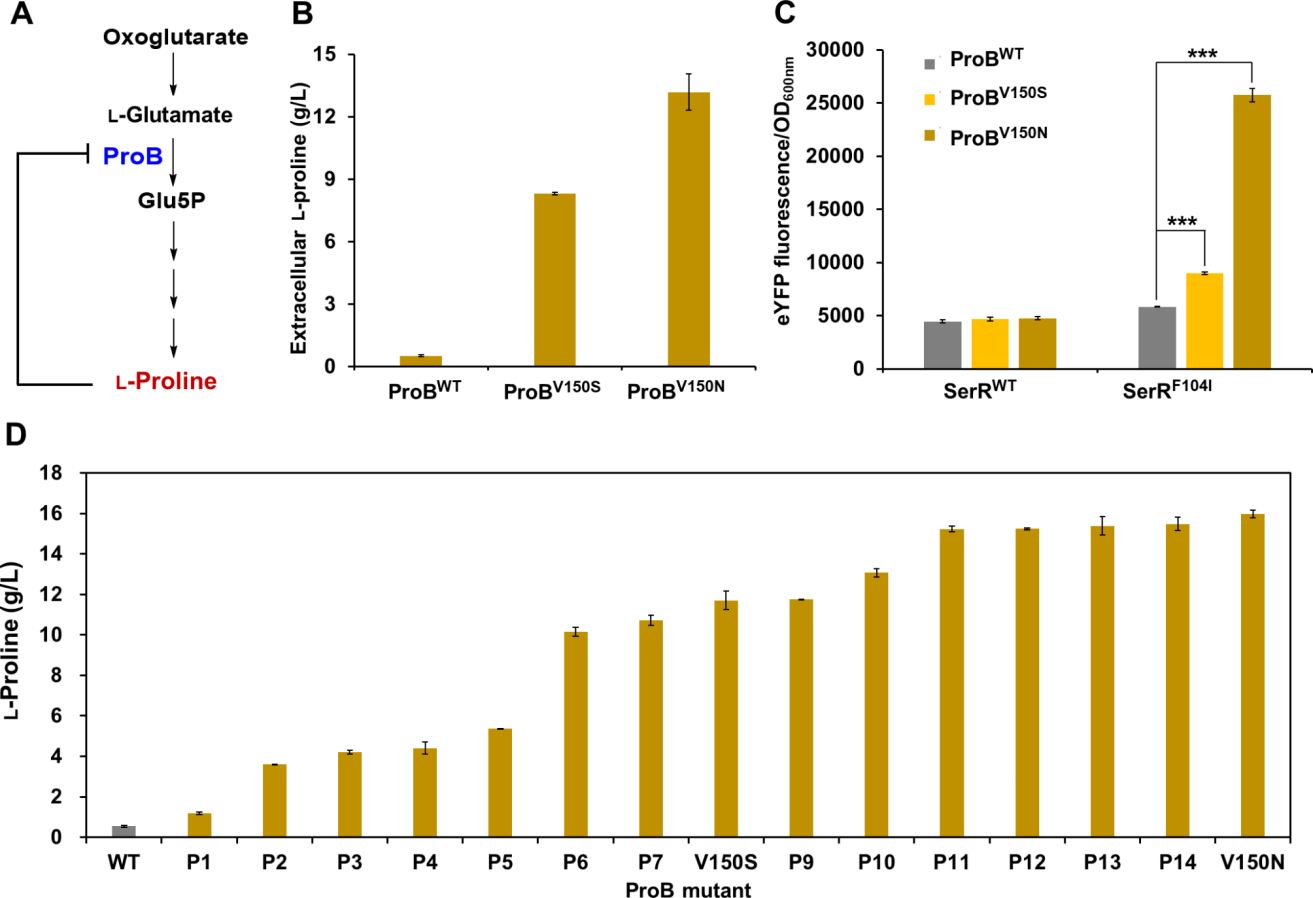
FACS-based screening of ProB mutants improving l-proline production. **(A)** Biosynthetic pathway of l-proline in *C. glutamicum*. The key enzyme γ-glutamyl kinase (ProB) and the target product l-proline are highlighted in blue and red, respectively. The feedback inhibition of ProB by l-proline is indicated with black line. **(B)** Extracellular accumulation of l-proline. ProB^WT^, ProB^V150S^, and ProB^V150N^ represent *C. glutamicum* transformed with the plasmid pXMJ19 overexpressing the wild-type ProB, ProB^V150S^, and ProB^V150N^, respectively. Extracellular l-proline titer was measured after 24 h cultivation in modified CGXII medium with 0.1 mM IPTG. **(C)** eYFP fluorescence signals of SerR^WT^- and SerR^F104I^-based biosensors with *C. glutamicum* strains overexpressing the wild-type ProB, ProB^V150S^, and ProB^V150N^. Strains were cultivated in modified CGXII medium with 0.1 mM IPTG for 12 h, and used for detection of the eYFP fluorescence. Values and error bars reflect the mean ± s.d. of three biological replicates (*n* = 3). ***P < 0.001, student’s two-tailed *t*-test. **(D)** Characterization of the isolated ProB mutants. The reported mutants ProB^V150S^ and ProB^V150N^ were used as positive controls. Strains were cultivated in modified CGXII medium with 0.1 mM IPTG for 24 h. Values and error bars reflect the mean ± s.d. of three biological replicates (*n* = 3)

Next, we constructed l-proline-producing *C. glutamicum* strains to test the l-proline biosensor based SerR^F104I^. l-Proline biosynthesis is tightly regulated by feedback inhibition of ProB by the end-product l-proline (Fig. 5A). Deregulation of the feedback inhibition of ProB facilitates l-proline production in *C. glutamicum* (Liu et al. 2022a; Zhang et al. 2020a). Therefore, the wild-type ProB and two mutants with deregulated feedback inhibition (ProB^V150S^ and ProB^V150N^) (Liu et al. 2022a) were overexpressed in plasmid pXMJ19, and transformed in wild-type *C. glutamicum* ATCC 13032, resulting in strains *C. glutamicum* (pXMJ19-*proB*^WT^), *C. glutamicum* (pXMJ19-*proB*^V150S^), and *C. glutamicum* (pXMJ19-*proB*^V150N^), respectively. The strain overexpressing the wild-type ProB on plasmid only produced 0.517 g/L l-proline, while overexpression of two reported mutants ProB^V150S^ and ProB^V150N^ (Liu et al. 2022a) with released feedback inhibition increased l-proline production by 16.1-fold (8.31 g/L) and 25.5-fold (13.2 g/L), respectively (Fig. 5B). The wild-type biosensor pSerR^WT^ cannot distinguish the strains with different l-proline production levels by outputting different eYFP signals (Fig. 5C). However, pSerR^F104I^ produced 1.37- and 4.68-fold higher eYFP outputs for ProB^V150S^ and ProB^V150N^ compared with the wild-type ProB, respectively (Fig. 5C). These results indicate that the l-proline biosensor pSerR^F104I^ could functionally respond to l-proline and could distinguish *C. glutamicum* strains producing different levels of l-proline.

Moreover, we used the whole-cell biosensor pSerR^F104I^ for high-throughput screening of ProB mutants enabling l-proline biosynthesis from a random mutation library. The procedure of ProB library construction and biosensor-based high-throughput screening was similar with that for directed evolution of Hom. Approximately 0.21% of cells with the highest fluorescence outputs in the library were sorted in the gate R5, while the portion for the wild-type ProB control was only 0.02%, suggesting ProB mutants enabling l-proline production existed in the library (Fig. S4). A total of 92 colonies sorted by FACS were selected and cultivated in 96-well plates for a second round of screening. Among them, 38 strains showed obviously higher eYFP fluorescence outputs than the control strain expressing wild-type ProB. Plasmid sequencing of the 38 strains revealed 13 kinds of ProB mutants with different amino acid substitutions, and the l-proline titers of these strains were all higher than that of the control strain (Fig. 5D). Four new mutants produced similar levels of l-proline with the best ProB^V150N^ mutant that we reported recently (Fig. 5D and Table S4) (Liu et al. 2022a).

Previous studies have demonstrated that the single-site mutation of residues A146, G149, and V150 of ProB contributed to the l-proline accumulation (Liu et al. 2022a; Zhang et al. 2020a). Interestingly, our recent study has demonstrated that single-site mutation of residue T148 or N151 did not enable the overproduction of l-proline (Liu et al. 2022a), while some ProB mutants with multiple-site amino acid residues mutation, such as P2 (N151D, A241T), P10 (P65T, N151S, G199A, I306T, N364Y), and P12 (A114T, T148A, N151S, D347V), did enhance the production of l-proline in our study. These results indicated that residue T148 (or residue N151) combined with other residues mutation were also important for the feedback regulation and catabolic activity of ProB.

These successful applications suggest that the engineered biosensor based on SerR^F104I^ is a useful tool for high-throughput screening of key enzymes involved in the biosynthesis of l-threonine and l-proline. Moreover, the biosensor also holds promise for high-throughput screening of hyper-producing strains of l-threonine and l-proline and dynamic regulation of the metabolic pathways (Liu et al. 2015; Wei et al. 2022). The SerR^F104I^-based biosensor simultaneously responds to l-serine, l-threonine, and l-proline. For certain application scenarios, biosensors specifically responding to one target effector may be needed. Therefore, engineering of the effector specificity of the biosensor by directed evolution or semi-rational design can be performed in the future (Della Corte et al. 2020; Liu et al. 2022b).

## Conclusions

In this study, inspired by the fact that amino acids sharing a transporter are usually recognized by the same transcriptional regulator, we selected the l-serine-responding transcriptional regulator SerR as the target for engineering of l-proline and l-threonine biosensors. Although the wild-type SerR only recognized l-serine, a single mutation F104I allowed SerR to recognize l-proline and l-threonine. An l-threonine and l-proline biosensor based on SerR^F104I^ was constructed for the first time and successfully employed to high-throughput screening of Hom and ProB mutants improving l-threonine and l-proline biosynthesis, respectively. This study provides a new strategy for engineering the naturally existing transcriptional regulators with expanded effector spectrum to develop whole-cell biosensors of amino acids or other chemicals.

## Materials and methods

### Bacterial strains and cultivation conditions

Bacterial strains used in this study can be found in Table S1. *E. coli* DH5α was used for cloning purposes and was cultivated aerobically in Luria–Bertani (LB) medium at 37 °C. Kanamycin (Km, 50 µg/mL), or chloramphenicol (Cm, 20 µg/mL) was added to the broth as required. *C. glutamicum* ATCC 13032 and its derivatives were cultivated aerobically at 30 °C in LBG broth (LB supplemented with 5 g/L glucose, pH 7.2) or TSB medium (Liu et al. 2022a). BHIS medium (38.5 g/L brain heart infusion, 91 g/L sorbitol, pH 7.2) with 3% (w/v) glycine and 0.1% (w/v) Tween 80 was used for the preparation of *C. glutamicum* competent cells (Wang et al. 2019a). Modified CGXII medium (Pu et al. 2023) was used as the fermentation medium for l-proline and l-threonine production. Where appropriate, Km (25 µg/mL), Cm (5 µg/mL), or IPTG (0.1 mM) was added in the medium. Genetic manipulation of *C. glutamicum*, e.g. preparation of competent cells, electroporation for transformation of strains, and gene deletion, were performed according to standard protocols (Liu et al. 2017a, 2018).

### Plasmid manipulation

All the plasmids and primers used in this study are listed in Tables S1 and S2, respectively. Plasmids were constructed via recombination using the ClonExpress MultiS One Step Cloning Kit (Vazyme, Nanjing, China). Services of primer and gene synthesis and DNA sequencing were provided by GENEWIZ Inc. (Suzhou, China). For the construction of plasmid pSerR^WT^, a fragment containing *serR* gene and *serE* promoter was amplified from genomic DNA of *C. glutamicum* ATCC 13032 using the primer pair *serR*-F/*serR*-R. The *eyfp* gene encoding eYFP protein was amplified from the plasmid pLysWT (Pu et al. 2023) with the primer pair *eyfp*-F/*eyfp*-R. The backbone of pTRCmob (Liu et al. 2007) was amplified by PCR with the primer pair pTRCmob-rev-F/pTRCmob-rev-R. Then *serR* gene with *serE* promoter and *eyfp* were ligated with the linearized pTRCmob through recombination, generating plasmid pSerR^WT^. The construction methods of other plasmids are the same as above.

### Generation of random mutation library

To generate the random mutation library of EBD region of SerR, error-prone PCR was conducted to produce different mutation rates of *serR* with the primer pair EBD-mut-F/EBD-mut-R using the genomic DNA of *C. glutamicum* ATCC 13032 as template through adding different concentrations of MnCl_2_ ranging from 0.05 mM to 5 mM. Then the error-prone PCR products were purified and recombined with plasmid pSerR^WT^, linearized by PCR with the primer pair EBD-Rev-F/EBD-Rev-R. Ligation products were transformed into *E. coli* DH5α competent cells. SerR mutation library plasmids, dubbed pSerR^mut^, were extracted from transformants, and then transformed into competent cells of *C. glutamicum* ATCC 13032 by electroporation. Construction of mutation libraries for other genes was conducted following the same procedure as above.

### FACS-based primary screening

For FACS-based sorting of the SerR mutation library, *C. glutamicum* strains were first cultivated in 1 mL LBG medium with 25 µg/mL Km in a 24-well plate at 800 rpm and 30 °C for 10 h in INFORS Microtron (INFORS HT Multitron Pro, Switzerland). Cells were used as seed cultures to inoculate 1 mL of modified CGXII medium with adding 0.4 M l-threonine or l-proline in 24-well plates to an optical density at 600 nm (OD_600nm_) of 0.5. After further cultivation for 6 h in INFORS Microtron, the cells were diluted to an OD_600nm_ below 0.1 with sterile phosphate buffered saline (PBS) and used immediately for FACS analysis. eYFP fluorescence was analyzed by flow cytometry (Beckman Coulter MoFlo XDP) with the method described by Pu, et al. (Pu et al. 2023). Clones of each gate showing an increased eYFP signal were sorted on LBG agar plates with 25 µg/mL Km, which were subsequently incubated for 24 h at 30 °C.

### Secondary screening in 96-well plates

Secondary screening of SerR mutants obtained from FACS were conducted in the 96-well plates. *C. glutamicum* strains pre-grown in LBG medium were inoculated into 96-well plates with 0.2 mL modified CGXII medium with or without 0.4 M l-threonine (or l-proline) to an OD_600nm_ of 0.5. After cultivation at 30 °C and shaking at 800 rpm for 12 h, cells were harvested and diluted properly with PBS. eYFP fluorescence intensities were determined using a microplate reader (SpectraMax M5, Molecular Devices, λ excitation = 488 nm, λ emission = 520 nm). The fluorescence intensities were normalized with OD_600nm_.

## Electronic supplementary material

Below is the link to the electronic supplementary material.

Supplementary Material 1: **Fig. S1**. Protein structure modeling of SerR. **Fig. S2**. The distance between the residue F104 and the effector l-serine. **Fig. S3**. Flow cytometry analysis of the random mutation library of Hom. **Fig. S4**. Flow cytometry analysis of the random mutation library of ProB. **Table S1**. Strains and plasmids used in this study. **Table S2**. Primers used in this study. **Table S3**. Characterization of Hom mutants. **Table S4.** Characterization of ProB mutants.

Supplementary Material 2

## Data Availability

The data and materials during the current study are available from the corresponding author upon reasonable request.

## Abbreviations

*C. glutamicum*: *Corynebacterium glutamicum*
FRET: Förster resonance energy transfer
LTTR: LysR-type transcriptional regulator
IPTG: Isopropyl-β-d-thiogalactopyranoside
eYFP: Enhanced yellow fluorescent protein
EBD: Effector binding domain
FACS: Fluorescence activated cell sorting
MD: Molecular dynamics
RMSF: Root mean square fluctuation
VMD: Visual molecular dynamics
HPLC: High-performance liquid chromatograph
LB: Luria-Bertani
Km: Kanamycin
Cm: Chloramphenicol
PBS: Phosphate buffered saline
