# Supplementary material for: Engineering of l-threonine and l-proline biosensors by directed evolution of transcriptional regulator SerR and application for high-throughput screening
Source: Bioresour Bioprocess. 2025 Jan 19;12(1):4. doi: 10.1186/s40643-024-00837-6 (PMC11743413; doi:10.1186/s40643-024-00837-6)
Supplement: Supplementary file 1 — Supplementary Material 1: Fig. S1. Protein structure modeling of SerR. Fig. S2. The distance between the residue F104 and the effector l-serine. Fig. S3. Flow cytometry analysis of the random mutation library of Hom. Fig. S4. Flow cytometry analysis of the random mutation library of ProB. Table S1. Strains and plasmids used in this study. Table S2. Primers used in this study. Table S3. Characterization of Hom mutants. Table S4. Characterization of ProB mutants. [file 40643_2024_837_MOESM1_ESM.docx]

**Supplementary information for**

**Engineering of l-threonine and l-proline biosensors by directed evolution of transcriptional regulator SerR and application for high-throughput screening**

**Wei Pu,^1,2,4,†^ Jinhui Feng,^1,4,†^ Jiuzhou Chen,^1,4,†^ Jiao Liu,^1,4^ Xuan Guo,^1,4^ Lixian Wang,^1,4^ Xiaojia Zhao,^1,5^ Ningyun Cai,^l,6^ Wenjuan Zhou,^1,4^ Yu Wang,^1,,3,4,5,*^ Ping Zheng,^1,4,5,*^ and Jibin Sun^1,4,5^**

^1^Key Laboratory of Engineering Biology for Low-carbon Manufacturing, Tianjin Institute of Industrial Biotechnology, Chinese Academy of Sciences, Tianjin 300308, China

^2^Key Laboratory of Regional Characteristic Agricultural Resources, College of Life Sciences, Neijiang Normal University, Neijiang 641100, China

^3^Haihe Laboratory of Synthetic Biology, Tianjin 300308, China

^4^National Center of Technology Innovation for Synthetic Biology, Tianjin 300308, China

^5^University of Chinese Academy of Sciences, Beijing 100049, China

^6^College of Biotechnology, Tianjin University of Science and Technology, Tianjin 300457, China

^†^These authors contributed equally to this work.

^*^Corresponding authors.


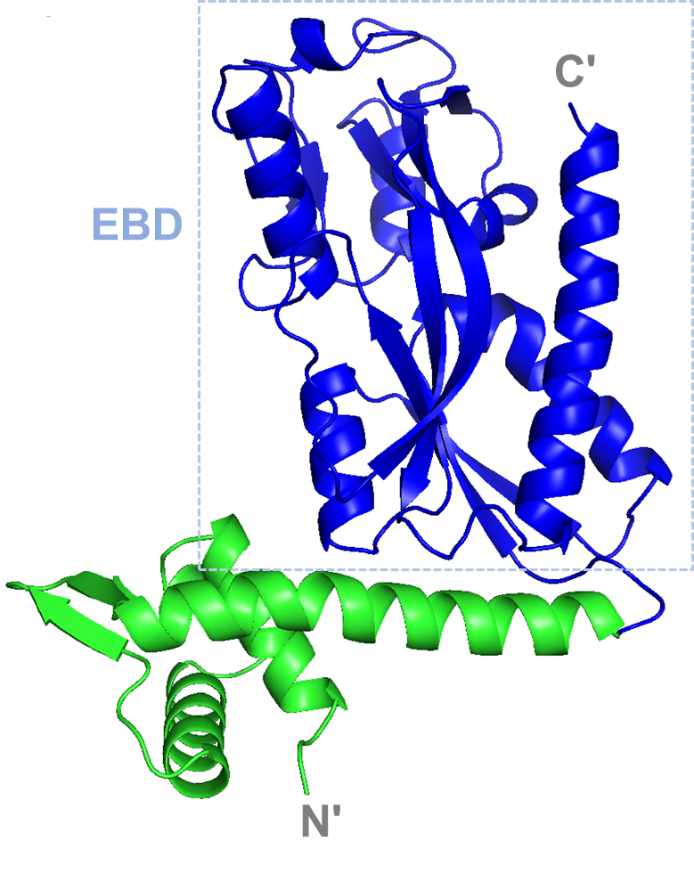


**Fig. S1.** Protein structure modeling of SerR. The structure of SerR was constructed based on the AlphaFold2. The effector binding domain (EBD) of SerR is highlighted in blue.


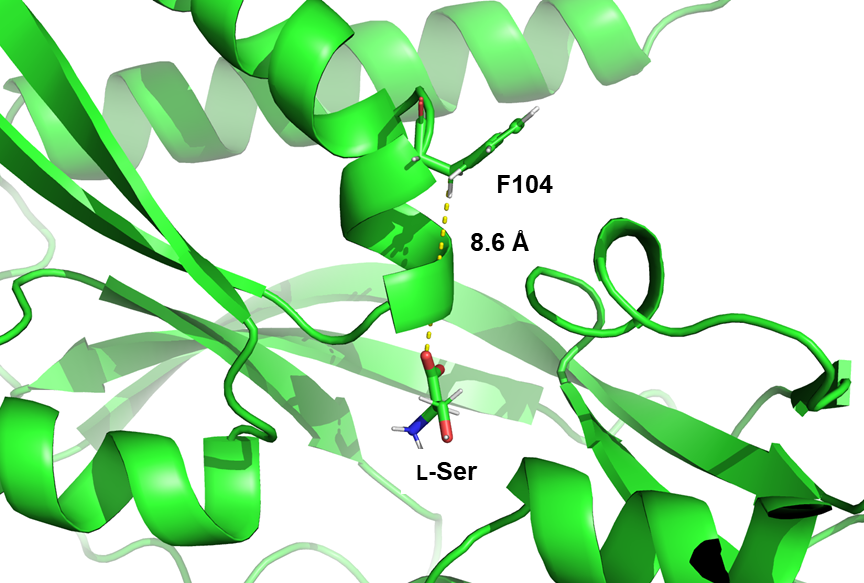


Fig. S2. The distance between the residue F104 and the effector l-serine. The residue F104 and effector l-serine were highlighted with bold black. The distance between residue F104 and effector l-serine was 8.6 Å.


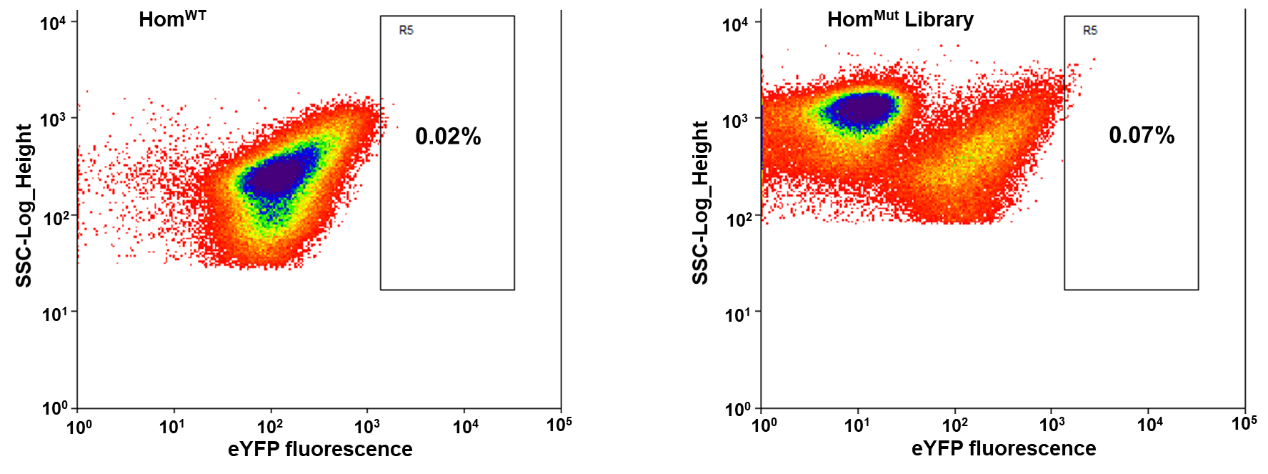


**Fig. S3** Flow cytometry analysis of the random mutation library of Hom. Hom^WT^, *C. glutamicum* Thr1 (pXMJ19-*hom*^WT^ + pSerR^F104I^-P_11F_-*thrB*). Hom^Mut^ Library, *C. glutamicum* Thr1 (pXMJ19-*hom*^Mut^ + pSerR^F104I^-P_11F_-*thrB*). Strains were cultivated in modified CGXⅡ medium with 0.1 mM IPTG, and subjected to FACS after 6 h cultivation.


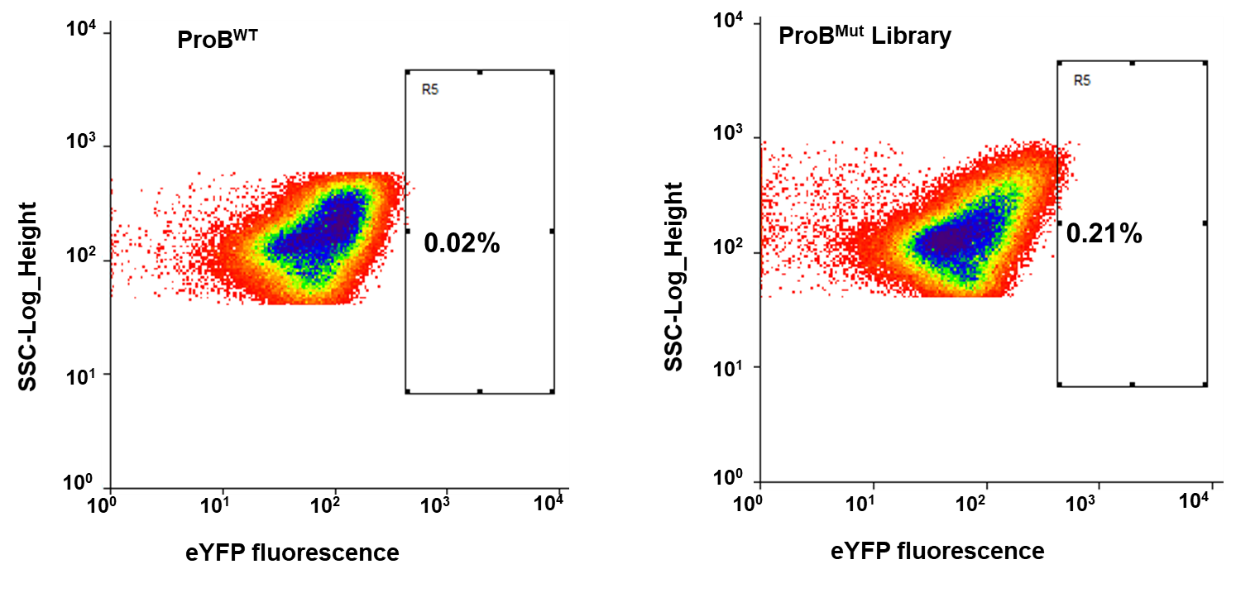


**Fig. S4** Flow cytometry analysis of the random mutation library of ProB. ProB^WT^, *C. glutamicum* (pXMJ19-*proB* + pSerR^F104I^). ProB^Mut^ Library, *C. glutamicum* (pXMJ19-*proB*^Mut^ + pSerR^F104I^). Strains were cultivated in modified CGXⅡ medium with 0.1 mM IPTG, and subjected to FACS after 6 h cultivation.

**Table S1.** Strains and plasmids used in this study

| **Strain or plasmid** | **Description** | **Reference or source** |
| --- | --- | --- |
| **Strain** |  |  |
| *E. coli* DH5α | F^−^ *supE*44 ∆*lacU*169 (φ80 *lacZ*∆M15) *hsdR*17 *recA*1 *endA*1 *gyrA*96 thi-1 *relA*1 | Invitrogen |
| *C. glutamicum* ATCC 13032 | Wild-type | Lab stock |
| LysC^I293Y^ | ATCC 13032 derivative with I293Y mutation of LysC | (Zheng et al. 2020) |
| Pro1 | ATCC 13032 derivative with G149K mutation of ProB | (Liu et al. 2022) |
| Thr1 | LysC^I293Y^ derivative with *hom* gene deleted | This study |
| 13032 (Δ*serE*) | ATCC 13032 derivative with *serE* gene deleted | This study |
| Pro1 (Δ*serE*) | Pro1 derivative with *serE* gene deleted | This study |
| Pro1 (Δ*thrE*) | Pro1 derivative with *thrE* gene deleted | This study |
| **Plasmid** |  |  |
| pXMJ19 | Expression vector of *C. glutamicum*, IPTG-inducible promoter P*_tac_*, Cm^R^ | (Jakoby et al. 1999) |
| pEC-XK99E | Expression vector of *C. glutamicum*, IPTG-inducible promoter P*_trc_*, Km^R^ | (Kirchner and Tauch 2003) |
| pTRCmob | Expression vector of *C. glutamicum*, IPTG-inducible promoter P*_trc_*, mob, Km^R^ | (Liu et al. 2007) |
| pSerR^WT^ | pTRCmob harboring *serR* and its target promoter fused to *eyfp* | This study |
| pSerR^F104I^ | pTRCmob harboring *serR*^F104I^ and its target promoter fused to *eyfp* | This study |
| pEC-XK99E-*thrE* | pEC-XK99E harboring *thrE* gene from *C. glutamicum* ATCC 13032 | (Liu et al. 2022) |
| pEC-XK99E-*serE* | pEC-XK99E harboring *serE* gene from *C. glutamicum* ATCC 13032 | This study |
| pXMJ19-*proB* | pXMJ19 harboring *proB* gene from *C. glutamicum* ATCC 13032 | This study |
| pXMJ19-*proB*^V150N^ | pXMJ19 harboring *proB*^V150N^ gene | This study |
| pXMJ19-*proB*^V150S^ | pXMJ19 harboring *proB*^V150S^ gene | This study |
| pXMJ19-*hom* | pXMJ19 harboring *hom* gene from *C. glutamicum* ATCC 13032 | This study |
| pXMJ19-*hom*^G378E^ | pXMJ19 harboring *hom*^G378E^ gene | This study |
| pSerR^WT^-P_11F_-*thrB* | pTRCmob harboring *serR*^WT^ and its target promoter fused to *eyfp,* and *thrB* gene driven by constitutive promoter P_11F_ | This study |
| pSerR^F104I^-P_11F_-*thrB* | pTRCmob harboring *serR*^F104I^ and its target promoter fused to *eyfp,* and *thrB* gene driven by constitutive promoter P_11F_ | This study |
| pCas9-gRNA-*ccdB* | pXMJ19 derivative, carrying *cas9* gene, driven by IPTG-inducible promoter P*_tac_* and *ccdB* gene, driven by constitutive promoter P_11F_ | (Liu et al. 2022) |
| pCas9-gRNA-Δ*hom* | pgRNA2 derivative carrying gRNA targeting *hom* and recombination arms for *hom* deletion | This study |
| pCas9-gRNA-Δ*thrE* | pgRNA2 derivative carrying gRNA targeting *thrE* and recombination arms for *thrE* deletion | (Liu et al. 2022) |
| pCas9-gRNA-Δ*serE* | pgRNA2 derivative carrying gRNA targeting *serE* and recombination arms for *serE* deletion | This study |

**Table S2.** Primers used in this study

| **Primer** | **Sequence (5’-3’)** | **Relevance** |
| --- | --- | --- |
| *serR*-F | GACATCATAACGGTTCTGGCTCACTCTACTAGACGAGCCTCCAA | pSerR^WT^ construction |
| *serR*-R | TCTTAAAGTTCATCTATTACGGTCCGATGGACAGTAAAAGA |  |
| *eyfp*-F | AACTTTAAGAAGGAGATATCATATGGTGAGCAAGGGCGAG |  |
| *eyfp*-R | TCTCATCCGCCAAAACAGCCTTACTTGTACAGCTCGTCCATGC |  |
| pTRCmob-rev-F | GGCTGTTTTGGCGGATGA |  |
| pTRCmob-rev-R | GCCAGAACCGTTATGATGTCG |  |
| *proB*-F | TAAGCTTGCATGCCTGCAGGAAGGAGATATACATATGCGTGAGCGCATCTCCAA | pXMJ19-*proB* construction |
| *proB*-R | ACCCGGGGATCCTCTAGAGTTTACGCGCGGCTGGCGTAGTT |  |
| pXMJ19-rev-F | ACTCTAGAGGATCCCCGGGTAC |  |
| pXMJ19-rev-R | CCTGCAGGCATGCAAGCTT |  |
| *hom*-F | TAAGCTTGCATGCCTGCAGGAAAGGAGTTGAGAATGACCTCAGCATCTGCCCCAA | pXMJ19-*hom* construction |
| *hom*-R | ACCCGGGGATCCTCTAGAGTTTAGTCCCTTTCGAGGCGGAT |  |
| *thrB*-F | AGAAGATTTTCAGCCTGATACTAAGGTTGGTTAACTTCAACCTTG | pSerR^F104I^-P_11F_-*thrB* construction |
| *thrB*-R | CTGCGTTCTGATTTAATCTGTTTTCTCCACATAAGCTGGCAATGTTGCGACGCAACAGGTACAGTGTAATTCAGACACACAGGAAACAGCTATGATGGCAATTGAACTGAACGT |  |
| pSerR-rev-F | CAGATTAAATCAGAACGCAGAAG |  |
| pSerR-rev-R | TATCAGGCTGAAAATCTTCTCTC |  |
| pEC-rev-F | TCTCAACTCCTTTGGCCTGTG | pEC-XK99E-*serE* construction |
| pEC-rev-R | CTGCAGGCATGCAAGCTTGG |  |
| *serE*-F | ACAGGCCAAAGGAGTTGAGATTGGCAATAATCAAGGGCAT |  |
| *serE*-R | CCAAGCTTGCATGCCTGCAGTTAACTAGGTGTGTGTACTC |  |
| Cas9-1 | TCGAAGGGCACCAATAACTGC | pCas9-gRNA-Δ*hom* construction |
| Cas9-2 | CTTTTACTTTCACCAGCGTTTCTG |  |
| Cas9-3 | AACGCTGGTGAAAGTAAAAGATGC |  |
| Cas9-4 | TCGCTTTCCACACCCGTGTTTGAATTACACTGTACCTGTTGCGTC |  |
| gRNA-1 | AACACGGGTGTGGAAAGCGAGTTTTAGAGCTAGAAATAG |  |
| gRNA-2 | CAACCTGCCATCACGAGATTTTC |  |
| *hom*-Left-1 | AATCTCGTGATGGCAGGTTGCACAATTTCTTTGCCCAGTTCG |  |
| *hom*-Left-2 | GCATCATCATCGCGCTCTTCGTACTCGGTCATCAGACGCATC |  |
| *hom-*Right-1 | GAAGAGCGCGATGATGATGC |  |
| *hom*-Right-2 | CAGTTATTGGTGCCCTTCGAGAAGCCGCAGCATTATCTGG |  |
| GK-1 | CCTACTCATGGGGACGCTACTAAG | Verification of *hom-*deleted strain |
| GK-2 | AAGGCGACAAGGTGCTGATG |  |
| gRNA-3 | TCTTTAAGGCTGTTCATCGCGTTTTAGAGCTAGAAATAGCAAG | pCas9-gRNA-Δ*serE* construction |
| gRNA-4 | CAACCTGCCATCACGAGATTTTC |  |
| *serE*-Left-1 | AATCTCGTGATGGCAGGTTGACTTGCAGGTGTGGGTATTTC |  |
| *serE*-Left-2 | CGTTCCAATGGCAGCACCAAA |  |
| *serE*-Right-1 | GGTGCTGCCATTGGAACGATTCTGCTCAGCCTCGAACC |  |
| *serE*-Right-2 | CAGTTATTGGTGCCCTTCGA+GTGATTACCTGGATGAGACCGG |  |
| EBD-mut-F | ATCATAACGGTTCTGGCTCA | EBD of serR mutant library construction |
| EBD-mut-R | GCCGTTCTAGATTCGCTGTC |  |
| EBD-Rev-F | GACAGCGAATCTAGAACGGC |  |
| EBD-Rev-R | TGAGCCAGAACCGTTATGAT |  |
| Hom-mut-F | TGCAGGAAAGGAGTTGAGAATG | Hom mutant library construction |
| Hom-mut-R | CCGGGGATCCTCTAGAGTTTAG |  |
| Hom-rev-F | AAACTCTAGAGGATCCCCGGGT |  |
| Hom-rev-R | CATTCTCAACTCCTTTCCTGCA |  |
| ProB-mut-F | AGGAAGGAGATATACATATG | ProB mutant library construction |
| ProB -mut-R | CGGGGATCCTCTAGAGTTTA |  |
| ProB -rev-F | TAAACTCTAGAGGATCCCCG |  |
| ProB -rev-R | CATATGTATATCTCCTTCCT |  |

**Table S3.** Characterization of Hom mutants

| Hom mutant | Amino acid substitution |
| --- | --- |
| H1 | A277T, N297D, V334A |
| H2 | A256V |
| H3 | V19D |
| H4 | F360I |
| H5 | A4V, V104A, N118D, R422H |
| H6 | M34T, A277T, A406V |
| H7 | D206G, H368L |
| H8 | T176S |
| H9 | M183V, I226T |
| H10 | T38A, P51S, S243C |
| H11 | P7S, V114A |
| H12 | E400G |
| H13 | A45E, D189N |
| H14 | A253P, H258R, R422H |
| H15 | A224T, R345C |
| H16 | N297S |
| H17 | S276L, E382G |
| H18 | A358T |
| H19 | A216T, V341M |
| H20 | S385N |
| H21 | T31A |
| H22 | D445G |
| H23 | H283Y, T365A |
| H24 | E134V, G174S, N318S, H342D |
| H25 | M371I |

**Table S4.** Characterization of ProB mutants

| ProB mutant | Amino acid substitution |
| --- | --- |
| P1 | Q42R |
| P2 | N151D, A241T |
| P3 | K78R, A81T, V145E, T238A, Y332F |
| P4 | E74G, I132T |
| P5 | N122S, V233M, E313V |
| P6 | T126I, E313V |
| P7 | K78E, T126A, K259E, Y272C |
| P9 | V87A, A225V, A273V |
| P10 | P65T, N151S, G199A, I306T, N364Y |
| P11 | V29A, A61T, G94A, A123V, V150A |
| P12 | A114T, T148A, N151S, D347V |
| P13 | A114V, H257Y, A277T, V341I |
| P14 | A120V, V150E |

**References**

Jakoby M, Ngouoto-Nkili CE, Burkovski A (1999) Construction and application of new *Corynebacterium glutamicum* vectors. Biotechnol Tech 13:437-441.

Kirchner O, Tauch A (2003) Tools for genetic engineering in the amino acid-producing bacterium *Corynebacterium glutamicum*. J Biotechnol 104:287-299.

Liu J, Liu M, Shi T, Sun G, Gao N, Zhao X, Guo X, Ni X, Yuan Q, Feng J, Liu Z, Guo Y, Chen J, Wang Y, Zheng P, Sun J (2022) CRISPR-assisted rational flux-tuning and arrayed CRISPRi screening of an L-proline exporter for L-proline hyperproduction. Nat Commun 13:891.

Liu Q, Ouyang SP, Kim J, Chen GQ (2007) The impact of PHB accumulation on L-glutamate production by recombinant *Corynebacterium glutamicum*. J Biotechnol 132:273-279.

Zheng P, Liu J, Wang Y, Zhou W, Sun J, Chen J, Ma Y, 2020. Polypeptide with aspartate kinase activity and use thereof in production of amino acid. Patent. p. CN113201514A, China.
