# Supplementary figures and images for: Engineering of l-threonine and l-proline biosensors by directed evolution of transcriptional regulator SerR and application for high-throughput screening
Source: Bioresour Bioprocess. 2025 Jan 19;12(1):4. doi: 10.1186/s40643-024-00837-6 (PMC11743413; doi:10.1186/s40643-024-00837-6)

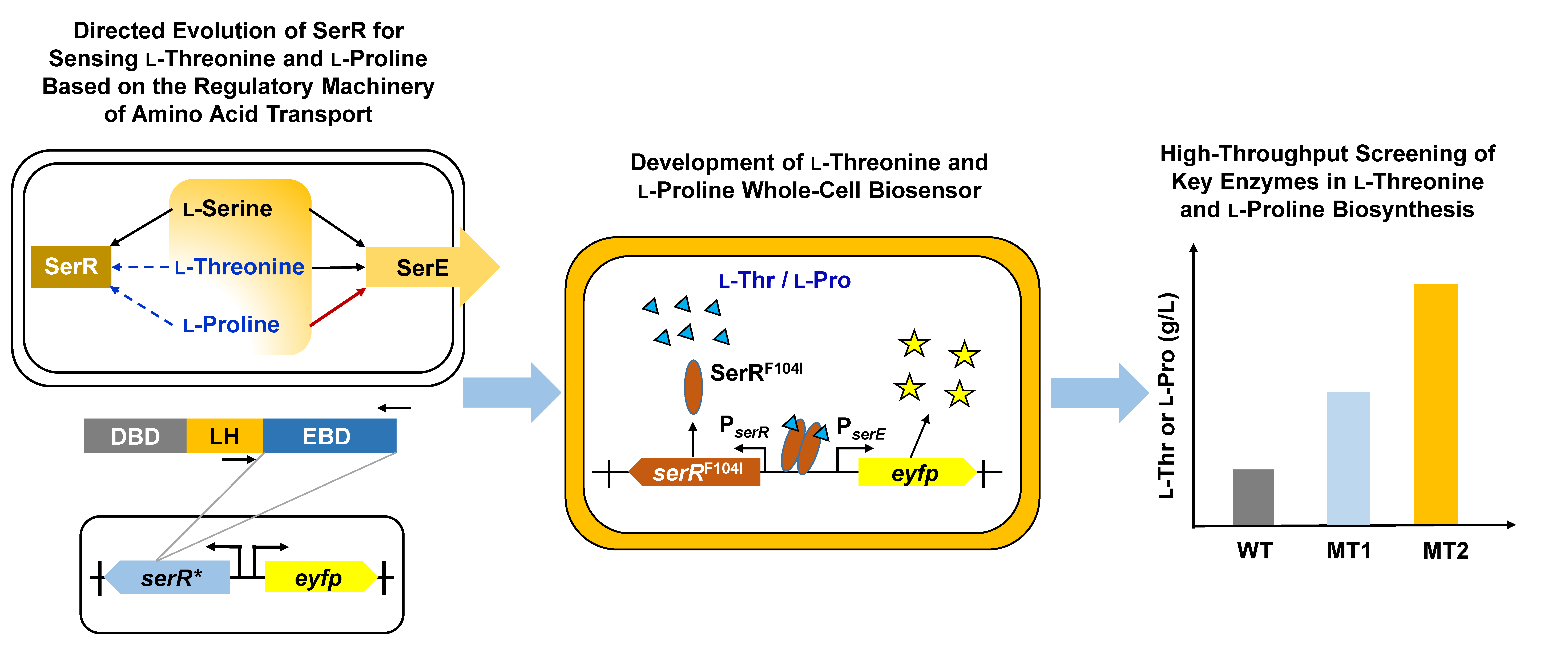

Supplement: Supplementary file 2 — Supplementary Material 2 [file 40643_2024_837_MOESM2_ESM.png]
